# Globin-like proteins in *Caenorhabditis elegans*: *in vivo *localization, ligand binding and structural properties

**DOI:** 10.1186/1471-2091-11-17

**Published:** 2010-04-02

**Authors:** Eva Geuens, David Hoogewijs, Marco Nardini, Evi Vinck, Alessandra Pesce, Laurent Kiger, Angela Fago, Lesley Tilleman, Sasha De Henau, Michael C Marden, Roy E Weber, Sabine Van Doorslaer, Jacques Vanfleteren, Luc Moens, Martino Bolognesi, Sylvia Dewilde

**Affiliations:** 1 Department of Biomedical Sciences, University of Antwerp, Universiteitsplein 1, B-2610 Antwerp, Belgium; 2 Institute of Physiology and Zürich Center for Integrative Human Physiology (ZIHP), University of Zürich, Winterthurerstrasse 190, CH-8057, Zürich, Switzerland; 3 Department of Biomolecular Sciences and Biotechnology, University of Milano, Via Celoria 26, I-20133 Milano, Italy; 4 Department of Physics, University of Antwerp, Universiteitsplein 1, B-2610 Antwerp, Belgium; 5 Department of Physics, University of Genova, Via Dodecaneso 33 I-16146 Genova, Italy; 6 INSERM U779, University of Paris 11, Hopital de Bicetre, Le Kremlin Bicêtre, 94275, France; 7 Zoophysiology, Department of Biological Sciences, Aarhus University, C F Møllers Allé, Bygning 1131, DK-8000 Aarhus C, Denmark; 8 Department of Biology, Ghent University, K L Ledeganckstraat 35, B-9000 Ghent, Belgium

## Abstract

**Background:**

The genome of the nematode *Caenorhabditis elegans *contains more than 30 putative globin genes that all are transcribed. Although their translated amino acid sequences fit the globin fold, a variety of amino-acid substitutions and extensions generate a wide structural diversity among the putative globins. No information is available on the physicochemical properties and the *in vivo *expression.

**Results:**

We expressed the globins in a bacterial system, characterized the purified proteins by optical and resonance Raman spectroscopy, measured the kinetics and equilibria of O_2 _binding and determined the crystal structure of GLB-1* (CysGH2 → Ser mutant). Furthermore, we studied the expression patterns of *glb-1 *(ZK637.13) and *glb-26 *(T22C1.2) in the worms using green fluorescent protein technology and measured alterations of their transcript abundances under hypoxic conditions.GLB-1* displays the classical three-over-three α-helical sandwich of vertebrate globins, assembled in a homodimer associated through facing E- and F-helices. Within the heme pocket the dioxygen molecule is stabilized by a hydrogen bonded network including TyrB10 and GlnE7.GLB-1 exhibits high ligand affinity, which is, however, lower than in other globins with the same distal TyrB10-GlnE7 amino-acid pair. In the absence of external ligands, the heme ferrous iron of GLB-26 is strongly hexacoordinated with HisE7, which could explain its extremely low affinity for CO. This globin oxidizes instantly to the ferric form in the presence of oxygen and is therefore incapable of reversible oxygen binding.

**Conclusion:**

The presented data indicate that GLB-1 and GLB-26 belong to two functionally-different globin classes.

## Background

The increasing availability of genomic data shows that globin-like proteins/domains (Mr ~17 kD) are universal and arose very early in the evolution of life [1]. Recent studies reveal new globin types as well as new functions (for a review see: [2-4]). A general survey suggests that the majority of nematodes host globin-like proteins [5]. Based on their tissue localisation, these proteins can be grouped in three classes: pseudocoelomic fluid, body wall, and cuticular hemoglobins (Hb). Some nematode species may however lack a particular class [5]. To date, the best characterized pseudocoelomic fluid Hb is the Hb from the ascarid *Ascaris suum*. This is an octameric molecule (Mr ~350 kDa) consisting of two layers of four subunits stacked in an eclipsed orientation [6]. Each subunit of 43 kDa consists of two covalently linked, highly similar globin domains (Mr ~17 kDa; 62% identical) followed by a C-terminal polar zipper of 23 amino acids. This Hb has an exceptionally high O_2 _affinity (K_O2 _= *k*_on_/*k*_off_: 0.215 M^-1^; P_50_: 0.001-0.004 torr at 20°C) that is attributed to a very slow dissociation rate (*k*_off_(O_2_): 0.0041 s^-1^) and a normal association rate (*k*_on_(O_2_): 1.5 μM^-1^s^-1^) [6], whereby this Hb will be in the oxy form even in the host's gut with a locally micro-oxygen concentration [6]. This high O_2 _affinity can be explained structurally by the presence of three hydrogen bonds between TyrB10 and GlnE7 and the bound ligand [7,8]. The function of the *A. suum *pseudocoelomic fluid Hb is still a matter of debate (for a review see: [5,6]). Due to its extremely high O_2 _affinity, it will rarely be deoxygenated *in vivo *and cannot therefore function as an O_2 _carrier or store. Several alternative functions have been proposed, such as O_2 _or NO scavengers, iron and/or heme stores, catalyst in sterol biosynthesis, buffering, osmotic - or other unknown functions [6,9].

The existence of globins in *C. elegans *was unexpected given the worm's small size, whereby sufficient amounts of O_2 _may reach the terminal oxidase sites by simple diffusion. The complete *C. elegans *genome revealed 33 globin-like genes, all having orthologues in the sibling species *C. briggsae *[10,11]. All 33 putative globin genes are expressed, albeit at low or very low levels, most likely indicating cell-specific expression. They show wide diversity in gene structure and amino-acid sequence of the translated proteins, suggesting a long evolutionary history. Nevertheless, sequence similarity to standard vertebrate globins, such as Hb α, β chains, myoglobin (Mb), neuro- and cyto-globin (Ngb and Cygb) can be detected [11], despite the presence of additional interhelical, N- and/or C-terminal extensions. The intron/exon patterns of these *C. elegans *globin-coding genes are unique in the number of introns and in their insertion positions, compared to the highly conserved intron/exon pattern of vertebrate and other non-vertebrate globin genes (B12.2 and G7.0) [11,12]. Given the large number of *C. elegans *globin genes it is unlikely that they all serve simple O_2 _metabolism. *C. elegans *is a soil dwelling nematode; soil parameters such as temperature, moisture and O_2 _concentration fluctuate, creating temporary hypoxic/anoxic environments [13]. Living systems have developed, at the organism and cellular levels, various strategies to cope with reduced O_2 _levels, *e.g*. increasing the glycolytic flux, reducing the aerobic metabolic rate and increasing the efficiency of O_2 _uptake from the environment. However, nematodes including *C. elegans *lack both specialized respiratory systems and complex circulatory organs, and rely on respiratory adjustments at the molecular levels [14,15].

Specialized O_2_-sensing cells in the nervous system of *C. elegans *permit rapid behavioural responses to O_2 _availability. The signal transduction pathway, detecting upper and lower O_2 _levels, involves the cGMP-gated channel *tax-2/tax-4 *and a soluble guanylate cyclase homologue, *gcy-35*. The expressed N-terminal domain, GCY-35(1-252) displays the spectral characteristics of a ferrous high-spin hemoprotein capable of O_2_, CO and NO binding [16]. Wild-type animals survive short periods of hypoxia/anoxia by greatly reducing metabolic rate and arresting movement and development. On the other hand the metabolic rate of the animals is not affected by increasing O_2 _levels up to 100% [17].

With the aim of shedding light on the roles played by *C. elegans *globins, we cloned several globin genes, and focussed our attention on two translation products (GLB-1 and GLB-26). We analyzed their expression patterns and alterations of transcript abundance under hypoxia. We then analyzed the two expressed globins through optical and resonance Raman spectroscopy, characterized the kinetics of ligand binding and O_2 _equilibrium properties, and determined the high resolution crystal structure of GLB-1. The results are discussed in the context of current views on multivariate globin function, of heme/ligand recognition mechanisms, and of the evolution of the globin fold through the phyla.

## Results

### Expression cloning of globins

Eight *C. elegans *globins were selected for cloning and expression. These were the canonical globin, GLB-1, three globins (GLB-14, GLB-18, GLB-29) that show sequence similarity to Cygb, two globins displaying similarity to Ngb (GLB-7, GLB-13), GLB-23 that contains an exceptionally large N-terminal extension, and GLB-26 in which Ile substitutes the highly conserved Phe at position CD1. All globin genes were cloned into pET3a and expressed *in vitro *in *E. coli*. After expression, only two globins, GLB-1 and GLB-26 were found in the cytosolic fractions with incorporated heme, whereas all the others were present as apo-proteins in inclusion bodies. All attempts to reconstruct the purified apo-proteins with hemin *in vitro *using different methods (see Methods and additional file 1 Table S1 and Figure S1) failed. As many globins are easily reconstructed from hemin and their apo-protein we concluded that GLB-7, GLB-13, GLB-14, GLB-18, GLB-23 and GLB-29 under the conditions used are unable to bind heme and/or properly fold into a native hemoprotein.

Based on previous experience with different globins, for crystallisation purposes, the Cys residues in GLB-1 were mutated to Ser. In GLB-1 Cys residues are present at positions A11, E15 and GH2. Mutation of CysA11 and/or CysE15 to Ser resulted in expression in inclusion bodies and in failure to incorporate hemin *in vitro *under the conditions used. In contrast, mutation of CysGH2 into Ser (GLB-1*) resulted in expression in the cytosolic fraction as a heme-containing globin. Inspection of the 3D structure of GLB-1* (see below) shows that the Cys residues at positions A11 and E15 are 9 Å apart and not linked by a disulphide bridge. The intrinsic incorrect folding displayed by the A11 and E15 Ser mutants suggests that the formation of a temporary disulfide bridge between these two residues is necessary during folding of the protein.

Gel filtration chromatography on a calibrated Superdex™ 75 column equilibrated in 50 mM Tris-HCl pH 8.5, 150 mM NaCl and 0.5 mM EDTA clearly shows that GLB-1, GLB-1* and GLB-26 behave in solution as non covalently bound dimers (data not shown).

### UV/VIS and Resonance Raman spectra

**Figure 1:**
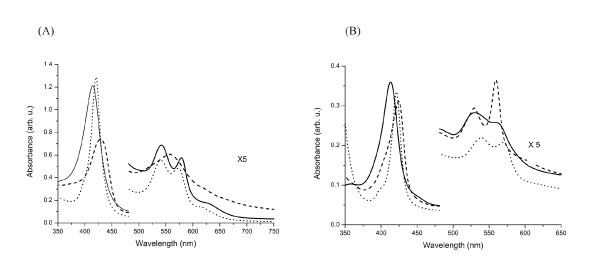
**UV/VIS spectra of different forms of (A) GLB-1 and (B) GLB-26**. Solid line: as-purified protein; dashed line: deoxy ferrous form; dotted line: CO-ligated ferrous form.

UV/VIS absorbance spectra of GLB-1 (as purified) shows the Soret band centered at 414 nm (Figure 1A); the *β*- and *α*- peaks at ~542 and ~580 nm, respectively, typical of an oxygenated globin. The shoulder at 635 nm indicates that a considerable fraction of a high-spin (HS) ferric species is also present. After deoxygenation and reduction, the Soret band shifts to 430 nm and the visible absorbance spectrum changes to that typical for the pentacoordinated HS ferrous form of globins. In the absorption spectrum of CO-ligated ferrous GLB-1, the Soret band peak at ~421 nm, and the *β*- and *α*-bands at ~540 and ~572 nm, respectively. The UV/VIS spectra of GLB-1* match those of w.t. GLB-1 (not shown).

**Figure 2:**
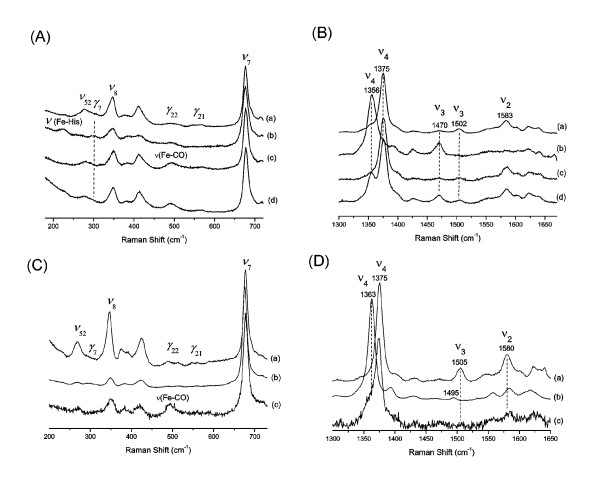
**RR spectra of GLB-1 and GLB-26**. (A) Low-frequency region and (B) high-frequency region (GLB-1) (a) As-purified form, laser power 30 mW (b) deoxy ferrous form, laser power 50 mW (c) CO form, laser power 2 mW, (d) CO form, laser power 40 mW. (C) Low-frequency region and (D) high-frequency region (GLB-26) (a), As-purified form, laser power 20 mW (b) deoxy ferrous form, laser power 25 mW (c) CO form, laser power 1 mW.

The high-frequency region of the RR spectrum of GLB-1 (as purified) is in agreement with the UV/VIS spectrum indicating a prevalence of the oxy form (*ν*_4_, *ν*_3_, and *ν*_2 _situated at ~1375 cm^-1^, ~1505 cm^-1^, and ~1580 cm^-1 ^respectively) (Figure 2B(a)). The weak line at 1470 cm^-1 ^indicates a small HS ferric fraction, in agreement with the UV/VIS spectra. After deoxygenation and reduction, the RR spectrum becomes typical of a (pentacoordinated) HS ferrous globin, again paralleling the UV/VIS data (*ν*_4_, and *ν*_3 _at ~1355 cm^-1^, and ~1470 cm^-1 ^respectively (Figure 2B(b)). Furthermore, the *ν*(Fe-His) stretching mode is observed at 225 cm^-1^, typical for HS globins (Figure 2A(b)). The RR spectra of GLB-1* matched closely those of GLB-1 (data not shown).

The RR spectrum of the CO-ligated form of GLB-1 displays *ν*_4_, *ν*_3_, and *ν*_2 _at ~1375 cm^-1^, ~1505 cm^-1^, and ~1585 cm^-1 ^respectively (Figure 2B(c)). Upon increase of the laser power, a second component appears in the RR spectrum (Figure 2B(d)). This component (*ν*_4 _~1356 cm^-1 ^and *ν*_3_~1470 cm^-1^) is typical of HS ferrous globins, indicating that photodissociation of CO has taken place. A strong peak around ~493 cm^-1 ^is observed that decreases upon strong photo-dissociation (not shown), indicative of the ν(Fe-CO) mode (Figure 2A(c-d)). Fe-CO stretching modes at higher frequencies cannot be observed. However, it should be noted that, since no isotopic labelling of the CO ligand was performed, weak Fe-CO stretching modes may escape attention and the current results should be interpreted cautiously.

The Soret band of GLB-26 (as purified) is found at 413 nm (Figure 1B), and the *β*- and *α*- bands display maxima at 530 and 565 nm, respectively, indicating that the globin is in a hexacoordinated low-spin (LS) ferric state. Upon reduction, the Soret band shifts to 424 nm, indicative of ferrous globins, with an intense α band at 560 nm indicating a hexacoordinated LS ferrous heme protein. The CO-ligated ferrous GLB-26 exhibits a typical UV/VIS spectrum, as also observed for CO-ligated GLB-1.

The RR spectra of GLB-26 (as purified) are typical of hexacoordinated LS ferric heme proteins (*ν*_4_, *ν*_3_, and *ν*_2 _at ~1375 cm^-1^, ~1505 cm^-1^, and ~1580 cm^-1^, respectively), in agreement with the UV/VIS data (Figure 2D(a)). In the low-frequency region, the out-of-plane mode *γ*_7 _is hardly detectable, indicating no significant out-of-plane distortion (Figure 2C(a)). After reduction of GLB-26, the RR spectra become typical for hexacoordinated LS ferrous globins (*ν*_4 _and *ν*_3_, at ~1364 cm^-1 ^and ~1495 cm^-1^, respectively) (Figure 2D(b)). In the RR spectrum of the CO-ligated ferrous form of GLB-26, the *ν*(Fe-CO) mode is again situated at ~493 cm^-1 ^(Figure 2C(c)). No clear *ν*(Fe-CO) modes were observed at higher frequencies.

### The 3D structural model of GLB-1*

The structure of GLB-1* (bearing the Cys(127)GH2 → Ser mutation to avoid protein aggregates during crystallization) was solved by MAD methods based on the anomalous signal of the heme iron atom. Diffraction data were collected at three wavelengths at the ID29 ESRF beamline (Grenoble, France), for the tetragonal *P*4_3_2_1_2 form (one GLB-1* molecule per asymmetric unit). Refinement of the crystal structure (at 1.5 Å resolution) converged at a general R_factor _value of 16.3% (R_free _of 20.1%), with ideal stereochemical parameters [18,19]. The final model contains 1,305 protein atoms (residues 2-159), 1 heme, 1 dioxygen molecule and 202 ordered solvent atoms (2 of them in alternate sites) (for details see additional file 2 Table S2). The tertiary structure of GLB-1* conforms to the three-over-three α-helical sandwich of the classical globin fold [20,21], where the seven/eight helices building up the globin fold are conventionally labelled, A, B, ..., H, according to their sequential order (topological sites are numbered sequentially within each helix; [22]). For comparison, a rmsd value of 1.79 Å is calculated (over 125 Cα atom pairs) in a structural overlay on sperm whale Mb, the largest structural deviation matching a 4-residue insertion at the AB loop, the CD interhelical region (one-residue insertion), a 3-residue insertion at the EF hinge, and a 2-residue deletion in the GH region. Residues 17-19 (the last turn of the A-helix), 43-48 (the C-helix) and 102-104 (the last turn of the F-helix) of GLB-1* display a 3_10 _conformation.

The diffraction data collected at 2.8 Å resolution on GLB-1* crystals grown in the trigonal *P*3_1_21 space group (two molecules per asymmetric unit) were also refined, yielding R_factor _and R_free _values of 26.9% and 31.9%, respectively, with ideal stereochemical parameters (for details additional file 2 Table S2). Superposition of 158 C_α _atoms of the two independent molecules, in the trigonal crystal form, yielded a rmsd of 0.53 Å. Comparison of the GLB-1* structures from the tetragonal and trigonal crystal forms yielded rmsd of 0.53 Å and 0.42 Å, for 158 C_α _atoms, depending on the chains superposed. The only backbone differences observed in the two crystal forms are located at the N- and C-terminal residues of the polypeptide chain (residues 2 and 159, respectively), and at residues 124-126 of the GH hinge.

**Figure 3:**
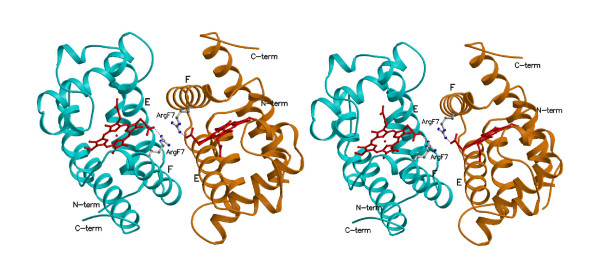
The GLB-1* dimeric assembly. A stereo view of the GLB-1* dimer. The two GLB-1* subunits, interacting mainly through the E and F helices, are shown in cyan and orange ribbon models. In GLB-1*P4_3_2_1_2 form the dimer sits on a crystallographic 2-fold axis, whereas in the *P*3_1_21 form one dimer is present in the crystallographic asymmetric unit. Figure drawn with Molscript [66].

All results discussed below apply to both tetragonal and trigonal crystal forms unless otherwise stated. In the high resolution tetragonal crystal form the crystallographic two-fold axis gives rise to a GLB-1* homodimer, whose 945 Å^2 ^association interface is mostly built by residues belonging to the E- and F helices (residues 72-83, and 87-101 respectively), and to the AB (residues 20-25) and EF (residues 84-86) corners of both chains. The crystallographic dimer is identical to the quaternary assembly found in the trigonal crystal form, where two crystallographically independent GLB-1* chains fill the asymmetric unit. The rmsd calculated over the whole dimer in the two crystal forms is 0.50 Å. The heme groups belonging to the two subunits lie 21 Å far apart (Fe to Fe distance). In particular, the D-propionates of each heme group are stabilized by an intramolecular salt bridge with the NH_2 _of residue Arg(100)F7 (2,77 Å), and can establish an intermolecular salt bridge with Arg(100)F7' belonging to the facing subunit (4,1 Å; Figure 3). The dimerization interface observed for the GLB-1 dimer is tightly packed, the stability of the assembly being granted by several hydrophobic interactions, hydrogen bonds (also mediated by water molecules and, in the trigonal crystal form, also by sulphate ions) and salt bridges. Such a highly symmetrical homodimer (all intermolecular interactions obey a 2-fold symmetry) is predicted to be energetically stable by the Protein Interfaces, Surfaces and Assemblies (PISA) detection software [23]. Remarkably, the GLB-1* dimeric assembly is strictly reminiscent of the quaternary assembly observed in *Scapharca inaequivalvis *Hb [24] and in *Caudina arenicola *Hb [25].

**Figure 4:**
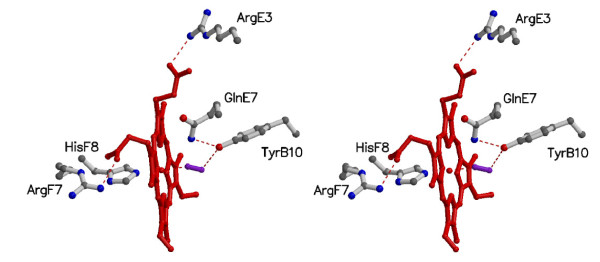
The heme site pocket of GLB-1*. Stereo view of the heme, of the proximal His(101)F8, of the distal Gln(69)E7 and of the surrounding residues contacting the heme. The O_2 _molecule is shown in ball-and-stick (violet). When appropriate, residue topological positions are indicated. Hydrogen bonds are dashed. Figure drawn with Molscript [66] (For the electron density maps see additional file 2, figure S2).

Stabilization of the heme group within the GLB-1* fold occurs through 95 van der Waals contacts (≤ 4.0 Å); moreover, salt links are present between residue Arg(65)E3 and heme A-propionate, and between residue Arg(100)F7 and heme D-propionate. These two Arg residues, together with Arg(72)E10, Lys(50)C7, Lys(68)E6, and Lys(105) build an evident positively charged patch around the heme crevice, mostly located on the heme distal side (Figure 4). The proximal His(101)F8 residue is properly coordinated to the heme-Fe atom (2.06 Å coordination bond), and is fully staggered relative to the porphyrin pyrrole N-atoms, allowing in principle a short Fe--HisF8 coordination bond, and in-plane location of the Fe atom. The HisF8 staggered orientation is defined by a strong hydrogen bond (2.83 Å) of His(101)F8 ND1 atom to the carbonyl O atom of residue Thr(97)F4.

Thanks to the high stability of the oxygenated form, the distal site of the crystallized GLB-1 hosts an O_2 _molecule coordinated to the heme-Fe atom (Fe --- O1 coordination distance 2.12 Å), adopting a moderately bent geometry (the Fe- O1-O2 angle is 155°C. The dioxygen molecule is stabilized, by a direct hydrogen bond (3.01 Å) to Tyr(35)B10, that in turn is hydrogen bonded (2.85 Å) to the NE2 atom of Gln(69)E7 (Figure 4). Tyr(35)B10 and Gln(69)E7 are the only polar residues in the heme distal site, which is essentially lined by aromatic residues (Phe(34)B7, Phe(38)B11, Phe(49)C6, Phe(66)E4, Ile(73)E11, and Phe(114)G8). Likely related to the overall apolarity, but also to the steric constraints posed by the above mentioned residues, no water molecules are observed in the heme distal site. The GLB-1* heme distal site resembles that of *A. suum *Hb, with TyrB10 directly stabilizing the O_2 _molecule and GlnE7 making a hydrogen bond with the B10 residue [7]. In *A. suum *Hb the coordination bond Fe---O1 is shorter (1.90 Å) than that found in GLB-1* and, due to the Arg → Phe residue substitution at the E3 topological site, the GLB-1* salt bridge to the heme A-propionate is not present in *A. suum *Hb.

### Ligand-binding: kinetic measurements and equilibrium data

**Figure 5:**
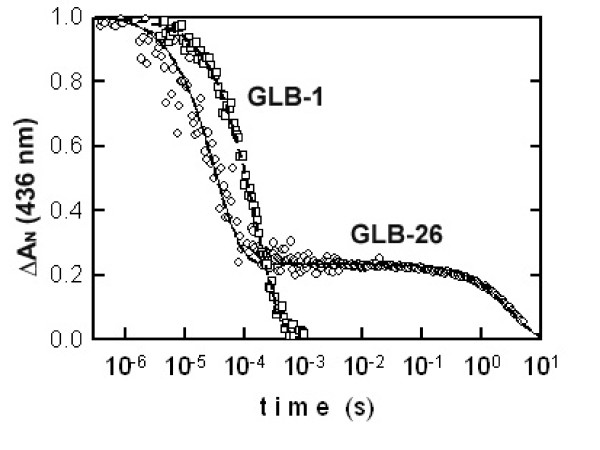
**Ligand rebinding kinetics after flash photolysis**. The kinetics of CO rebinding is monophasic for the globin GLB-1 as expected for a pentacoordinate heme. The kinetics for GLB-26 is biphasic and follows a simple model of competition for heme rebinding between a distal residue and CO.

**Figure 6:**
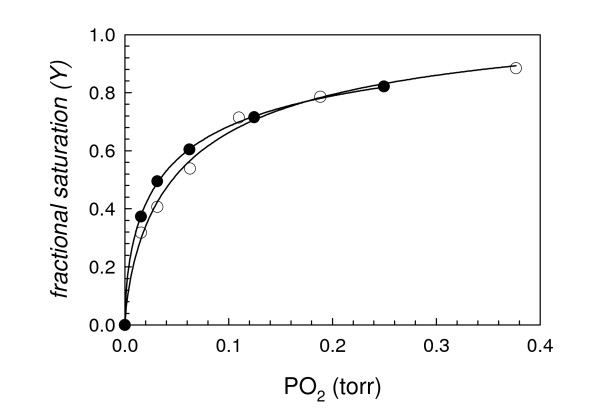
**O_2 _equilibrium curves of GLB-1 at pH 6.90 (open symbols) and pH 7.54 (closed symbols)**. Data fitting according to the Hill equation is shown. Solvent conditions: 100 mM Hepes buffer, 0.5 mM EDTA + Hayashi enzymatic reduction system [40].

The ligand-binding characteristics for both globins were measured using flash photolysis (Table 1). For GLB-1, the CO-rebinding kinetics are monophasic (Figure 5) and the k_off _for O_2 _is two orders of magnitude lower than for Mb, resulting in a high O_2 _affinity (46 μM^-1^, Table 1). Accordingly, O_2_-equilibrium experiments show that the O_2 _affinity of GLB-1 is high and pH-independent (Table 1, Figure 6), with P_50 _values of 0.062 ± 0.023 and 0.047 ± 0.007 torr at pH 6.90 and 7.54, respectively. Hill coefficients below unity (0.75 and 0.61 at pH 6.90 and 7.54, respectively) indicate that the subunits of this dimeric globin have different O_2 _affinity (here functional heterogeneity seems to apply only to O_2 _binding as CO kinetics are monophasic).

**Table 1:** Rates of ligand binding of *C. elegans *globins compared to rates reported for other globins.

		*C.elegans* GLB-1	*C.elegans* GLB-26	*H.sapiens* NGB	Sperm whale Mb	*A.suum* Hb
**O_2_**	**k_on_** **μM^-1^s^-1^**	23	n.d.	250	14	3
	
	**k_off_** **s^-1^**	0.5	n.d	0.8	12	0.013
	
	**K = k_on_/k_off_** **μM^-1^**	46	n.d	313	1.1	215
	
	**P_50_** **torr**	0.003	n.d	1	1	0.0072

**His**	**k_on_** **s^-1^**	-	20000	-	-	-
	
	**k_off_** **s^-1^**	-	0.3	-	-	-
	
	**K = k_on_/k_off_**	-	66667	-	-	-

**CO**	**k_on_** **μM^-1^s^-1^**	-	23	65	0.5	0.21
	
	**k_off_** **s^-1^**	-	0.05	0.01	0.02	0.018
	
	**K = k_on_/k_off_** **μM^-1^**	-	460	4643	28	12

(values for *H. sapiens *Ngb and sperm whale Mb from ref [67] and for *A. suum *Hb from ref [68])

n.d.: not detectable

Note: K_O2_, _obs _= K_O2, penta_/(1+ K_His_) = (k_O2, on_/k_O2, off_)/(1 + k_His, on_)/k_His, off_)

The same experiments were performed for GLB-26. It was difficult to measure the kinetics of GLB-26, because this globin is readily oxidized to the ferric species in the presence of O_2_. Several reducing systems were tried, but even then it was not possible to keep the heme iron of this protein reduced in the presence of O_2_. However, GLB-26 is easily reduced anaerobically by sodium dithionite or by NADPH under light exposure.

**Figure 7:**
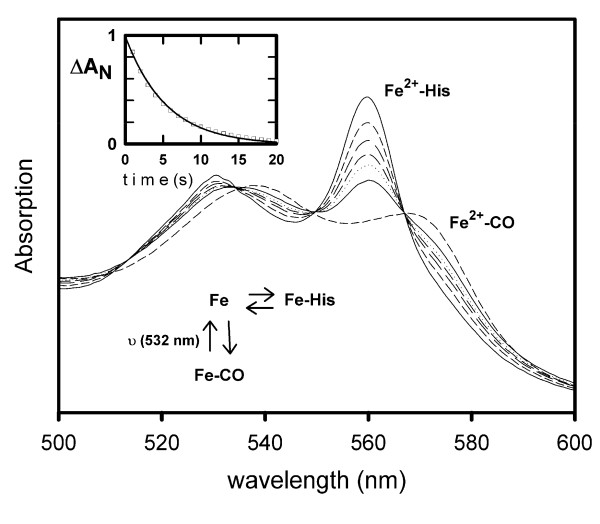
**His to CO replacement reaction of GLB-26**. The variation of absorption that occurs on the timescale of seconds was recorded in the visible region with a diode-array spectrophotometer. For the series of spectra, a decrease in absorbance at 560 nm is observed for increasing times. The observed spectral change is the same as the difference between the static spectra for the His and CO bound forms; this unambiguously assigns the slow transition after CO photo-dissociation (Figure 6) to the replacement of the internal His residue by CO. The insert shows the progression of the reaction, presented as the normalized fraction of the His bound form (the remainder in the CO bound form).

The CO-rebinding kinetics to the hexacoordinated GLB-26 are characterized by a biphasic trace (Figure 5) with a slow phase of replacement due to the slow dissociation of the distal residue. The characterization of this slow phase was confirmed by measurement of the full absorption spectra versus time (Figure 7). Based on the k_on _for the CO ligand, the k_on_-value for the distal ligand His was calculated to be 20 000 s^-1^.

In order to attempt to measure the O_2_-dissociation rate of GLB-26, mixed atmosphere O_2_/CO experiments were performed. The oxy complex of GLB-26 is unstable (millisecond timescale). After CO dissociation, a first phase of rebinding was measured where three ligands are in competition (O_2_, CO and His). A slow phase (5 s^-1^) was measured at equimolar ligand concentration (~500 μM O_2_/CO). This phase does not correspond to the O_2 _replacement by CO, but rather to the O_2_-induced oxidation of the heme, because the initial CO-globin signal (before flash photolysis) decreases after each rebinding cycle. Although a small fraction of transient O_2_-heme complex is replaced by CO, the k_off _(O_2_) is undistinguishable from that of the oxidation rate. O_2 _equilibrium measurements were tentatively run in the presence of the Hayashi reducing system. Even then, only ferric and deoxy globins were detected when changing O_2 _tensions, confirming that a stable oxy-derivative is not formed (Figure 6).

### In situ localisation

**Figure 8:**
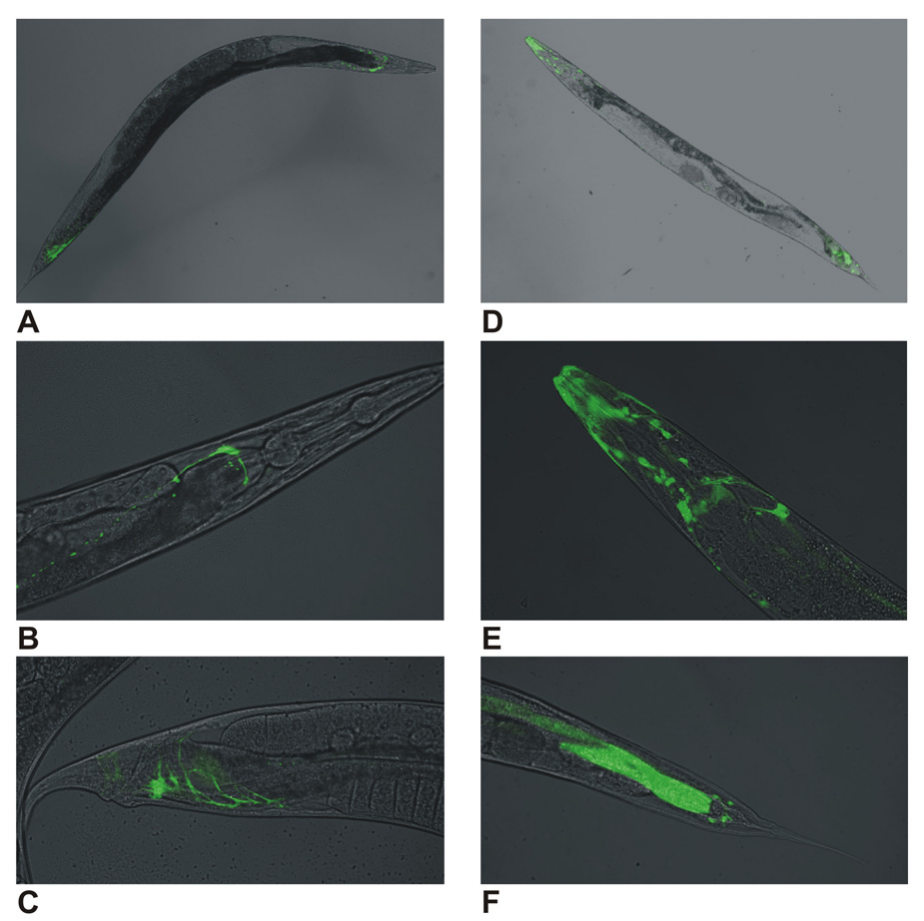
**Expression of globinpromotor::GFP fusions**. (A) *glb-26*::GFP expression, (B) *glb-26*::GFP expression in head mesodermal cell, (C) *glb-26*::GFP expression in stomato-intestinal muscle, (D) *glb-1*::GFP expression, (E) *glb-1*::GFP expression in the head, (F) *glb-1*::GFP expression in the tail, background fluorescence is also visible in the posterior intestine.

Analysis of tissue-specific expression patterns revealed that *glb-26 *is expressed exclusively in the head mesodermal cell (Figure 8A and 8B) and in the stomato-intestinal muscle (Figure 8A and 8C). The function of the head mesodermal cell is currently unknown. The stomato-intestinal muscle connects the surfaces of the posterior intestinal cells to the ventral epidermis and is coupled to the anal sphincter and anal depressor muscles via gap junctions. Contraction of these muscles promotes defecation. *glb-1 *is expressed mainly in a subset of neuronal cells and in head muscular tissue (Figure 8D, E and 8F). Both globin genes clearly display distinct expression patterns. Notably, the majority of *C. elegans *globins are expressed in the nervous system in highly cell-specific patterns [26].

### Globin expression under hypoxic conditions

To investigate hypoxic response, worms were subjected to hypoxia (0.1% oxygen) for 12 h in a hypoxic chamber, and globin gene expression levels were determined by quantitative real-time RT-PCR. Analysis of the expression profiles revealed significant upregulation for *glb-1 *(1.72; P < 0.01) whereas expression levels of *glb-26 *(0.93) remain unaffected by hypoxia (additional file 3 Figure S3).

## Discussion

Gas exchange in *C. elegans *relies completely on diffusion from the surrounding environment into the tissues [14,15]. *C. elegans *has an extended network of 302 neurons. Neuronal signal transduction consumes high amounts of energy, which mainly originates from aerobic metabolism. Neurons have therefore developed various adaptation strategies against hypoxic stress, for example the expression of globin-like proteins. *C. elegans *globin-like proteins are mainly present in neurons. It is therefore possible that *C. elegans *globin-like proteins play a role in the defence against the toxic effects of hypoxia/anoxia by functioning in detoxification of ROS and RNS as well as in O_2 _sensing and/or that they may have a role in redox reactions, rather than functioning as O_2 _carriers, as proposed for Ngb [27,28].

Although the *C. elegans *genome codes for more than 30 globin-like proteins, which are all expressed as demonstrated by RT-PCR, no characteristic UV/VIS globin absorption spectrum can be detected in a crude *C. elegans *extract. This suggests that the overall *in vivo *protein concentrations are very low [11], which tallies with the possibility that some of the globin-like molecules (e.g. *glb-1*) are expressed only in a small subset of cells.

Expression of a selected set of *C. elegans *globins in an *E. coli *system *in vitro*, demonstrates that, under the conditions used, only GLB-1 and GLB-26 can be recovered as hemoproteins in the cytosolic fraction of the cell. Refolding of the others, starting from the apoprotein, purified from inclusion bodies, and adding hemin *in vitro *failed. When inspecting the sequences of the *C. elegans *globins, a correlation was found between the presence of long N- and/or C-terminal extensions and the expression in inclusion bodies as was also seen previously for Cygb [29]. The inability to refold several of the globin-like proteins may also suggest that some of these may not incorporate a heme group *in vivo*. Hence, they could support a function that does not require heme. Such consideration, however, does not exclude that these proteins may play a physiological role. Indeed, the N-terminal domain of RsbR, a protein of the bacterial stressosomes (large signalling complexes that cope with environmental stress signalling) clearly displays the globin fold but is unable to bind heme [30]. Moreover, the N-terminal domain of RsbR dimerizes in a fashion identical to that of the globin-coupled sensor, HemAT of *Bacillus subtilis *and *Geobacter sulfurreducens *[31]. It is hypothezised that in RsbR the globin-folded domain evolved from an ancestral sensor globin related to the globin-coupled sensors, but lost its ability to bind heme in later evolutionary stages [3,30].

The UV/VIS and RR spectra of GLB-1 indicate that this globin is pentacoordinated in the deoxy form. The *ν*(Fe-CO) stretching mode, observed at ~493 cm^-1 ^has previously been observed in other globins, *e.g*. sperm whale Mb (491 cm^-1^) [32]. In Mb, this conformer becomes dominant at low pH values, when the distal HisE7 residue swings out of the heme pocket. In GLB-1 it is ascribed to an open conformation of the heme pocket, with a weak interaction between CO and the surrounding residues. No Fe-CO stretching modes at higher frequencies can be observed, in agreement with earlier observations on *Paramecium caudatum *Hb [33]. However, it should be noted that, since no isotopic labelling of the CO ligand was performed, weak Fe-CO stretching modes may escape attention. Unlike the CO form, the 3-D structure shows a stabilization of bound O_2 _through a hydrogen bond with TyrB10. Same is seen for *A suum *Hb [34].

GLB-1 displays a quite high O_2 _affinity, mainly due to the slow k_off _for O_2_. Because of the presence of the GlnE7/TyrB10 heme distal pair, a high O_2 _affinity was expected. Nevertheless, the O_2 _affinity is almost 5 times lower than that of *A. suum *(46 μM^-1 ^versus 215 μM^-1^). This difference is mainly due to a slower O_2 _dissociation process in *A. suum *Hb (0.013 s^-1^) than in GLB-1 (0.05 s^-1^), related to strong stabilization of the bound O_2 _by two hydrogen bonds, a short one with TyrB10 (2.73 Å) and a weaker one with GlnE7 (3.30Å) [6]. In GLB-1 TyrB10 also stabilizes the bound oxygen (3.01 Å), but the Fe---O1 coordination bond is longer than that in *A. suum *Hb. Therefore it is easier to break, resulting in a lower O_2 _affinity compared with *A. suum *Hb. The nemertean *C. lacteus *mini-Hb also displays a TyrB10/GlnE7 pair, but displays high rates of O_2 _dissociation (*k*_*off*_*(*O_2_) ~200-600 s^-1^) and hence a moderate O_2 _affinity (*K*_O2_~1 μM^-1^) as a result of a third polar residue, ThrE11, in its distal site. In fact, the presence of Thr at position E11 modulates the formation of a hydrogen bond between TyrB10 and the heme-bound O_2 _[35].

UV/VIS spectra similar to those of ferrous GLB-26 were observed for the ferrous forms of Ngb [36], and Cygb [37]. The spectra are typical of ferrous globins exhibiting bis-histidine heme coordination. The *ν*(Fe-CO) stretching mode of CO-ligated ferrous GLB-26 is situated at ~493 cm^-1^, again typical of an open conformation of the heme pocket. No clear *ν*(Fe-CO) modes were observed at higher frequencies, in contrast to other heme proteins exhibiting bis-histidine coordination, such as Ngb, which exhibits three intense Fe-CO stretching modes (one open and two closed conformations) [37].

The biphasic plot observed for the GLB-26 CO-rebinding kinetics results from the competition between CO and the distal HisE7 ligand. The slow phase of replacement indicates that the dissociation of the distal E7 residue is slow. For most hexacoordinated globin samples equilibrated under 1 atm CO, this phase is only a few percent. The calculated k_on _value for the distal ligand His (20000 s^-1^) is the highest ever measured for a hexacoordinated globin (one order of magnitude larger than for Ngb) [36,38]. Also note that the K_CO _of GLB-26 is about one order of magnitude lower than that of Ngb (Table 1). This is in agreement with the RR results indicating an open conformation of the GLB-26 CO-ligated form. In Ngb, closed conformations have been identified [37].

Concerning the rates of O_2_-binding, both equilibrium and flash photolysis data confirm that GLB-26 is not capable of reversible O_2 _binding, and hence cannot act as an O_2 _store. Other globins, like Ngb, Cygb, 2-on-2 (truncated) hemoglobins and globin-coupled sensors, clearly illustrate different possible functions for members of the globin superfamily [3,39,40]. The tissue distribution of Ngb, Cygb and the hypoxia-inducible myoglobin expression in non-muscle tissues in fish supports the hypothesis that globins may occur in every cell [41].

Additional *in vivo *analysis underscores the different behaviour of GLB-1 and GLB-26. GFP reporter assays revealed a clearly distinct expression pattern of *glb-1 *and *glb-26*. In addition both globins behave differently under hypoxic conditions (0.1% O_2_); *glb-1 *is significantly upregulated under hypoxia, whereas expression of *glb-26 *remains unchanged. Strikingly, the opposite was observed under conditions of total anoxia; whereas the expression level of *glb-1 *is unaffected, expression of *glb-26 *is induced.

## Conclusion

GLB-1 and GLB-26 are fundamentally different relative to their tissue localisation, expression pattern under hypoxia and anoxia, ligand-binding mechanism and affinities. Ferrous GLB-1 binds O_2 _with a high affinity and is pentacoordinated in the absence of exogenous heme ligands. Taken together, the observed functional data suggest for GLB-1 a putative role *in vivo *related to O_2 _binding, NO scavenging or O_2 _storage. This globin could serve to maintain the O_2 _concentration at a constant level provided that its cellular concentration is high enough. The induced GLB-1 expression under hypoxia conditions is consistent with a possible role in oxygen-dependent metabolism. Similar hypoxia-dependent inductions have been reported for globins putatively involved in oxygen transport or storage [42,43]. Interestingly, several hypoxia-tolerant organisms including *Daphnia, Danio rerio *and Carassius auratus display upregulation of pentacoordinated globins upon hypoxic exposure [44-47]. Furthermore, the oxygenated globin could play a role in regulating NO-homeostasis.

Ferrous GLB-26 globin cannot serve such functions due to its inability to form a stable oxygenated species. It can be speculated that at high O_2 _levels GLB-26 will readily reduce O_2_; the reduced species may then participate in redox reactions or simply dismutate into O_2 _and H_2_O_2_. GLB-26 may thus resemble oxidases. Under anaerobic conditions, this globin might participate in other redox reactions. Indeed, the very high GLB-26 heme affinity for the distal HisE7 residue points to roles other than binding gaseous ligands. Interestingly, an N-terminal myristoylation site is predicted http://expasy.org/ with very high confidence (98.2%) for GLB-26, which may indicate membrane anchoring, an unprecedented globin function [48].

## Methods

### Recombinant expression of *C. elegans *globins

*C. elegans *worms were grown as described previously [10]. Young adult worms were collected and total RNA was prepared using the TriZol method (Invitrogen) followed by LiCl precipitation (Ambion). The cDNA of the *C. elegans *globins was prepared using the OneStep RT-PCR kit (Qiagen) and gene-specific primers (Eurogentec). Cycling conditions were as followed: 30 min at 50°C for the RT reaction, followed by 15 min at 95°C for the activation of the HotStar Taq DNA polymerase, followed by 35 cycles of 60 sec at 94°C, 60 s at 54°C and 90 s at 72°C. The cDNA was cloned into the pET3a vector (Stratagene) using *NdeI *and *BamHI *restriction enzymes (Biolabs, Westburg) and T4 Ligase (Novagen).

Mutations (Cys → Ser) were introduced in GLB-1 using the QuickChange™ site-directed mutagenesis method (Stratagene) as described previously [36]. The mutant bearing the CysGH2 → Ser substitution is annotated as GLB-1*.

Cloning and expression of *C. elegans *globins were performed as described previously [36]. Briefly, the expression plasmids were transformed into *Escherichia coli *strain BL21(DE3)pLysS (Invitrogen). Cells were grown at 25°C in TB medium containing 200 μg/ml ampicillin, 30 μg/ml chloramphenicol and 1 mM δ-amino-levulinic acid. The culture was induced at A_550 _= 0.8 OD with IPTG (final concentration 0.04 mM).

### Purification of recombinant *C. elegans *globins

After overnight growth, *E. coli *cells were collected. Recombinant GLB-1 and GLB-26 were spectroscopically localised in the cytosolic fraction. GLB-1 was purified to homogeneity using (i) ammonium sulphate precipitation (40%-90% saturation) after which the 90% pellet was dissolved and dialyzed against 5 mM Tris-HCl pH 8.5, (ii) DEAE-Sepharose fast flow chromatography (step elution in 5 mM Tris-HCl pH 8.5, 200 mM NaCl) and (iii) Sephacryl S200 gel filtration in 50 mM Tris-HCl pH 8.5, 150 mM NaCl, 0.5 mM EDTA. The globin fractions were pooled and concentrated.

GLB-26 was purified using (i) ammonium sulphate precipitation (60% saturation), (ii) CM-sepharose fast-flow chromatography (50 mM sodium phosphate buffer pH 7, step elution in 50 mM sodium phosphate buffer pH 7, 0-300 mM NaCl) and (iii) S-Sepharose column (50 mM sodium phosphate buffer, step elution in 50 mM sodium phosphate buffer pH 7, 0-500 mM NaCl).

After expression, *C. elegans *globins, GLB-7, GLB-13, GLB-14, GLB-18, GLB-23 and GLB-29 were localised as apo-protein in inclusion bodies. The standard purification procedure was as follows: cells were suspended in 50 mM Tris-HCl pH 7.5, 0.5 M NaCl, 1 mM EDTA, 1 mM phenylmethylsulfonylfluorid and 5 mM dithiotreitol. After suspension, 1/10 volume of 10% triton X-100, 10% deoxycholic acid, 500 mM Tris-HCl pH 7.5, 20 mM EDTA was added and the cells were exposed to three freeze-thaw cycles and sonication until complete lysis. Inclusion bodies were isolated by centrifugation at 3,300 g for 10 min and the pellet was suspended in and washed three times with 1% triton X-100, 1 mM EDTA and 50 mM Tris-HCl pH 7.5. Inclusion bodies were solubilized in 6 M guanidinium hydrochloride, 50 mM Tris-HCl pH 7.5 and 1% 2-mercaptoethanol for 1 hr at 0°C. After elimination of the insoluble material by centrifugation (10 min 10,000 g), the *C. elegans *globins were refolded by adding a 1.4 M excess of hemin and dialysis against 5 mM Tris-HCl pH 8.5 at 4°C. The refolded *C. elegans *globins were further purified using Sephacryl S200 gelfiltration in 50 mM Tris-HCl, pH 8.5, 150 mM NaCl, 0.5 mM EDTA.

When no heme was incorporated into the *C. elegans *globins by this approach, other methods were tried. For details see additional file 4.

### Optical and Resonance Raman spectroscopy

UV/VIS spectroscopy was performed on a Cary-5 UV/VIS-NIR spectrophotometer. All optical spectra were measured in a 200-800 nm range. Resonance Raman (RR) measurements were carried out on an 80-cm Dilor XY-800 Raman scattering spectrometer consisting of a triple spectrograph operating in normal mode and a liquid nitrogen-cooled CCD detector. The excitation source was a Kr-ion laser (Spectra Physics 2020) at 413.1 nm. The protein solution was stirred at 6,000 rpm to avoid local heating. Five spectra (120-s recording time) were acquired and averaged after the removal of cosmic ray spikes by a program developed in-house. Laser powers of 1 and 50 milliWatt were used. For UV/VIS RR spectroscopy, the CO-ligated ferrous forms of the *C. elegans *globins were prepared by adding an excess of sodium dithionite and subsequently passing the sample through a PD10 column (Amersham Biosciences) equilibrated with CO-flushed Tris-HCl buffer (5 mM, pH 8.5). The deoxy ferrous form was obtained by equilibration under nitrogen and by addition of an excess of sodium dithionite. The concentration of the protein samples used for optical and RR measurements was typically ~60 μM in Tris-HCl buffer (5 mM, pH 8.5).

### Crystallization and X-ray Data Collection

Crystallization of GLB-1* was achieved using the hanging dropvapour diffusion setup. In the material used for crystallization the Fe^2+^-O_2 _form is the predominant form. Using the fully oxygenated and oxidized spectra of Mb as reference, a deconvolution of the UV/Vis spectra resulted in 30% (± 5%) ferric form and 70% (± 5%) Fe^2+^-O_2 _form. The GLB-1* solution, at 35 mg/ml, was equilibrated against a precipitant solution containing 3.0 M ammonium sulphate and 10% glycerol (v/v), at 277 K. Rod-like crystals grew in about 2-3 weeks. The crystals were transferred in a solution containing 3.3 M ammonium sulphate and 20% glycerol (v/v) immediately prior to data collection (at 100 K). These crystals diffracted up to 2.8 Å resolution using synchrotron radiation (beamline ID14-3, ESRF, Grenoble, France; trigonal space group *P*3_1_21 (or enantiomorph), with unit cell parameters: *a *= *b = *77.7 Å, *c *= 145.6 Å, two GLB-1* molecules per asymmetric unit).

In order to obtain better quality crystals, a wider screen (566 in-house-designed conditions) was set up, using the sitting dropvapour diffusion method and a robotic apparatus (Genesis RSP100 - Tecan). Large single crystals grew within 2-3 weeks using PEG 4 k 10%, 0.1 M sodium acetate (pH 5.5) at 277 K. They were transferred in a solution containing 30% PEG 4 k, 0.1 M sodium acetate (pH 5.5), and 15% (v/v) glycerol, immediately prior to cryo-cooling and data collection. A three-wavelength multi-wavelength anomalous dispersion (MAD) data set was collected at ESRF synchrotron (beamline ID29, Grenoble, France) at 100 K. The peak and inflection point wavelengths were determined by collecting an X-ray absorption spectrum near the heme iron atom K absorption edge. The crystals diffracted up to 1.5 Å resolution (remote wavelength data set) and belong to the tetragonal *P*4_3_2_1_2 space group (or enantiomorph), with unit cell parameters: *a *= *b = *81.9 Å, *c *= 47.0 Å (one GLB-1* molecule per asymmetric unit). All collected data were reduced and scaled using MOSFLM and SCALA, respectively [49,50]. Data collection and processing statistics are reported in additional file 2.

### Structure Determination and Refinement

MAD phases, based on the heme-Fe atom anomalous signal, were determined on the tetragonal crystal form (space group *P*4_3_2_1_2) at 1.9 Å resolution with SOLVE [51] with a figure of merit of 0.37. The electron density map was improved by solvent flattening with DM [52] yielding a figure of merit of 0.86. ARP/wARP [53] was used to extend and refine phases to 1.5 Å resolution, and for automated model building of all the main and side chain atoms. The molecular model was subsequently checked manually with COOT [53,54] and refined to the maximum resolution (1.5 Å) using REFMAC [55]. At the end of the refinement stages (including anisotropic B-factor refinement), a heme-bound dioxygen and 202 solvent molecules were located through inspection of difference Fourier maps.

The refined structure of the GLB-1* monomer was then used as a starting model to solve the structure of the trigonal crystal form using the program MOLREP [56]. The rotational and translational searches yielded two prominent solutions in the 34.3-2.8 Å resolution range for space group *P*3_1_21. Initially the two GLB-1* molecules were rigid-body refined using the program REFMAC [55]. At the end of the restrained refinement cycles 46 water molecules were located through the inspection of difference Fourier maps, using the program COOT [54].

The programs Procheck and Surfnet [19,57] were used to assess stereochemical quality and to explore protein matrix cavities. The program PISA [23] was used to analyse quaternary assemblies within the crystal unit cell. Atomic coordinates and structure factors have been deposited with the Protein Data Bank [58] with entry codes 2 wtg and r2wtgsf (1.5 Å resolution) and 2wth and r2wthsf (2.8 Å resolution), respectively.

### Ligand binding

#### Kinetic measurements

Ligand-binding kinetic measurements were performed by laser photodissociation as described elsewhere [59]. Samples were equilibrated under air or 0.1 atm or 1 atm CO. For oxygen dissociation rates (k_off_), the ligand replacement reaction was use: a mixed CO/O_2 _atmosphere was used; photodissociation of CO allows association of oxygen followed by a return to the CO form. Kinetics were measured and recorded at various wavelengths alternatively on a LeCroy oscilloscope for microsecond to second timescales and a diode-array HP8453 spectrophotometer for longer times. The experiments were performed in a 50 mM potassium phosphate, 0.1 mM EDTA buffer at pH 7.0 and 25°C.

#### Equilibrium experiments

Ferric *C. elegans *globin samples were reduced anaerobically by dialysis against CO-equilibrated 50 mM Hepes buffer, 0.5 mM EDTA, pH 7.6, containing 2 mg/ml sodium dithionite and DTT as described [40] and stored in aliquots at -80°C as CO-derivative. Samples were thawed shortly before measurements and kept on ice until analysed.

O_2 _equilibrium curves of 3-μl samples of GLB-1 were recorded at 20°C by monitoring absorbance at 436 nm using a thin-layer equilibration chamber fed by cascaded Wösthoff gas mixing pumps that deliver a constant flow of precise mixtures of air or O_2 _and ultrapure (>99.998%) N_2_[60]. Samples were dissolved in 0.1 M Hepes buffer, 0.5 mM EDTA at a protein concentration of 0.3 mM heme, and contained the enzymatic met-Hb reducing system as previously detailed [40,61]. Before determination of O_2 _equilibria, CO was removed from the heme by repeated cycles of N_2_/O_2 _equilibration within the chamber until the absorbance difference between N_2 _and O_2 _equilibrated samples remained constant.

### Expression analysis under hypoxia

For hypoxia treatments, plates containing synchronized young adult worms were placed either in room air (normoxia control) or in a hypoxic chamber (MIC-101, Billups-Rothenberg Inc.) with constant gas flow. Worms were incubated for 12 h in 21% oxygen or 0.1% oxygen at 20°C. Animals were quickly harvested in S-buffer (43.55 mM KH2PO4, 6.45 mM K2HPO4 and 100 mM NaCl in distilled water, pH 6), RNA was extracted using the RNeasy Midi kit (Qiagen) according to the manufacturer's instructions. All samples were treated with DNase (Zymo Research). A NanoDrop ND 1000 spectrophotometer (Isogen) was used to analyze RNA concentration and purity. First strand cDNA was synthesized from 2 μg RNA using an oligo(dT) primer and Moloney murine leukemia virus reverse transcriptase (Fermentas) at 42°C for 1 hr. Quantitative RT-PCR was carried out using a Rotor-Gene 2000 centrifugal real-time cycler (Corbett Research) using the Platinum SYBR Green qPCR SuperMix-UDG (Invitrogen) as described previously [62]. A single melt peak for each reaction confirmed the identity of each PCR product. The threshold cycle (Ct) values of the Rotor-Gene software version 6.0 (Corbett Research) were exported to qBase version 1.3.5 [63] for further analysis. All measurements were produced in duplicate, and for each primer set, reaction efficiency estimates were derived from standard curves that were generated using serial dilutions of a cDNA pool of normoxic and hypoxic nematode samples. These were then used by qBase to transform the Ct values to relative quantities that were normalized using the geometric mean of three reference genes (*tba-1*, *csq-1 *and *cdc-2*) identified by the geNorm 3.4 software from a set of 8 candidate control genes [64]. The significance of data was evaluated by Student's t-test.

### Expression patterns

5' upstream sequences from *glb-26 *and *glb-1 *were extracted from the UCSC Genome Browser database using a repeat masker function http://genome.ucsc.edu. Putative promoters were derived from wild-type N2 genomic DNA by PCR. Promoter::green fluorescent protein (GFP) fusion constructs were made as described by Hobert [65]. In this procedure two primary PCR products, the globin promoter (1108 bp for *glb-26*, 2294 bp for *glb-1*) and the GFP encoding sequence plus the 3'UTR from *unc-54 *amplified from a fire's vector pPD95.75, are fused in frame in a subsequent fusion PCR employing nested primers.

Microinjection was carried out by injecting DNA into the gonads of young adult hermaphrodites using an AxioVert 135 (Zeiss) microscope and FemtoJet microinjection system (Eppendorf). For each promoter-GFP fusion construct 20 to 30 wild-type N2 worms were injected. The pRF4 plasmid was co-injected with the promoter-reporter fusion construct at concentrations of 100 ng/μl and 50 ng/μl, respectively. pRF4 contains the dominant marker *rol-6(su1006) *which confers a rolling phenotype allowing easy identification of transformed worms. Rolling first generation (F1) progeny were picked after four days of incubation at 20°C and checked for rolling F2 progeny after a further three- to four-day incubation period. At least 3 independent transgenic lines were examined for both globin genes. For imaging, worms were mounted on agarose pads and immobilized with 10 mM sodium azide. Images were taken using a D-Eclipse C1 Confocal Microscope (Nikon).

### Accession numbers

Atomic coordinates and structure factors have been deposited with the Protein Data Bank [58] with entry codes 2 wtg, and r2wtgsf (1.5 Å resolution), and 2wth, and r2wthsf (2.8 Å resolution), respectively.

## Abbreviations

Hb: hemoglobin; Mb: myoglobin; Ngb: neuroglobin; Cygb: cytoglobin; RR: Resonance Raman; MAD: Multi-wavelength anomalous dispersion; rmsd: root mean square deviations; GFP: green fluorescent protein; HS: High spin; LS: Low spin; CM-sepharose: Carboxy methyl sepharose; S-sepharose: sulphopropyl sepharose; NIR: near infra red; 'as purified': refers to globin samples obtained after purification without additional modification in ligand binding.

## Authors' contributions

EG carried out the expression cloning with help of LT, purified the globins and drafted the manuscript; DH and SDH generated fusion constructs, performed microinjection experiments and analyzed globin expression levels under hypoxic conditions. MN, AP and MB carried out the 3D-structural determination of GLB-1*. EV and SVD carried out the UV-VIS spectroscopy and Resonance Raman measurements. AF and RW carried out the equilibrium measurements, MM and LK the kinetic measurements. LM, JV and SD were responsible for the study design and coordination of it. All authors read and approved the final document.

## Supplementary Material

Additional file 1 **Characteristics of the *C. elegans *globins studied**. A table with all characteristics of the globins studied and an allignement of all globin sequences of *C.elegans*. Click here for file

Additional file 2 Data collection and refinement statistics for GLB-1*. Contains the data collection and refinement statistics and the stereochemical analysis of GLB-1* crystal structure. Click here for file

Additional file 3 **Globin expression levels under hypoxic conditions**. Graph of globin expression levels under hypoxic conditions. Click here for file

Additional file 4 **Purification of Recombinant *C. elegans *globins**. detailed protocol for the purification of all studied globins. Click here for file
