# Supplementary material for: Globin-like proteins in Caenorhabditis elegans: in vivo localization, ligand binding and structural properties
Source: BMC Biochem. 2010 Apr 2;11:17. doi: 10.1186/1471-2091-11-17 (PMC2867796; doi:10.1186/1471-2091-11-17)
Supplement: Additional file 1 — Characteristics of the C. elegans globins studied. A table with all characteristics of the globins studied and an allignement of all globin sequences of C.elegans. [file 1471-2091-11-17-S1.DOC]

Additional file 1:

Characteristics of the *C. elegans* globins studied

Table S1

**Figure S1: Alignment of the *C. elegans* globins cloned in the pET3a vector** Full information about these globins can be found in [www.wormbase.org](http://www.wormbase.org/)
