# Supplementary material for: Globin-like proteins in Caenorhabditis elegans: in vivo localization, ligand binding and structural properties
Source: BMC Biochem. 2010 Apr 2;11:17. doi: 10.1186/1471-2091-11-17 (PMC2867796; doi:10.1186/1471-2091-11-17)
Supplement: Additional file 2 — Data collection and refinement statistics for GLB-1*. Contains the data collection and refinement statistics and the stereochemical analysis of GLB-1* crystal structure. [file 1471-2091-11-17-S2.DOC]

Additional file 2

Data collection and refinement statistics for GLB-1*

*Data collection statistics*

| Space group | *P*4_3_2_1_2  Inflection point | *P*4_3_2_1_2  Absorption edge | *P*4_3_2_1_2  Remote | *P*3_1_21 |
| --- | --- | --- | --- | --- |
| Cell dimensions (Å) |  |  | *a* = 81.9  *b =* 81.9  *c* = 47.0 | *a* = 77.7  *b =* 77.7  *c* = 145.6 |
| Resolution limits (Å) | 57.93-2.0  (2.11-2.00)^a^ | 57.93-2.0  (2.11-2.00) | 41.0-1.5  (1.58-1.50) | 67.3 -2.8  (2.95-2.8) |
| Observations | 147033  (21043) | 225538  (32491) | 716706  (104711) | 115384  (17000) |
| Unique reflections | 10790  (1489) | 10825  (1503) | 25649  (3623) | 12777  (1829) |
| Completeness (%) overall | 95.9  (93.3) | 96.1  (93.7) | 98.0  (96.8) | 98.4  (99.0) |
| R-merge*^b^* (%) | 10.9  (72.6) | 11.0  (71.7) | 5.6  (27.9) | 6.5  (32.1) |
| I/σ(I) | 22.3  (3.6) | 26.7  (4.7) | 50.4  (13.0) | 23.3  (7.5) |
| Multiplicity | 13.6  (14.1) | 20.8  (21.6) | 27.9  (28.9) | 9.0  (9.3) |

*^a^* The outer shell statistics are included in parentheses.

*^b^* R-merge =Σ_h_Σ_i_ | I_hi_ – <I_h_> | / Σ_h_Σ_i_ I_hi_.

*Refinement statistics and stereochemical analysis*

| *Refinement statistics:* | *P*4_3_2_1_2 | *P*3_1_21 |
| --- | --- | --- |
| Resolution range (Å) | 36.6-1.5 | 34.3 -2.8 |
| R-factor*^a^*/R-free*^b^* (%) | 16.3/20.1 | 26.9/31.9 |
| Number of residues :  monomer A  monomer B | 158  --- | 158  158 |
| Number of heme groups | 1 | 2 |
| Number of oxygen molecules | 1 | 2 |
| Number of sulfate ions | -- | 3 |
| Number of glycerol molecules | -- | 6 |
| Number of water molecules | 202 | 46 |
| *Stereochemical analysis:* |  |  |
| R.m.s. deviation from ideality:  bond lengths (Å)  bond angles (°) | 0.012  1.431 | 0.011  0.996 |
| Ramachandran plot: |  |  |
| Residues in most favored regions (%) | 96.5 | 91.0 |
| Residues in additional allowed regions (%)  Residues in generously allowed regions (%) | 3.5  -- | 8.7  0.3 |

*^a^* R-factor = Σ_h_ ||F_obs_| - |F_calc_|| / Σ |F_obs_| where F_obs_ and F_calc_ are the observed and calculated structure factor amplitudes, respectively.

*^b^* R-free is calculated with 10% of the diffraction data, which were not used during the refinement.

**A**


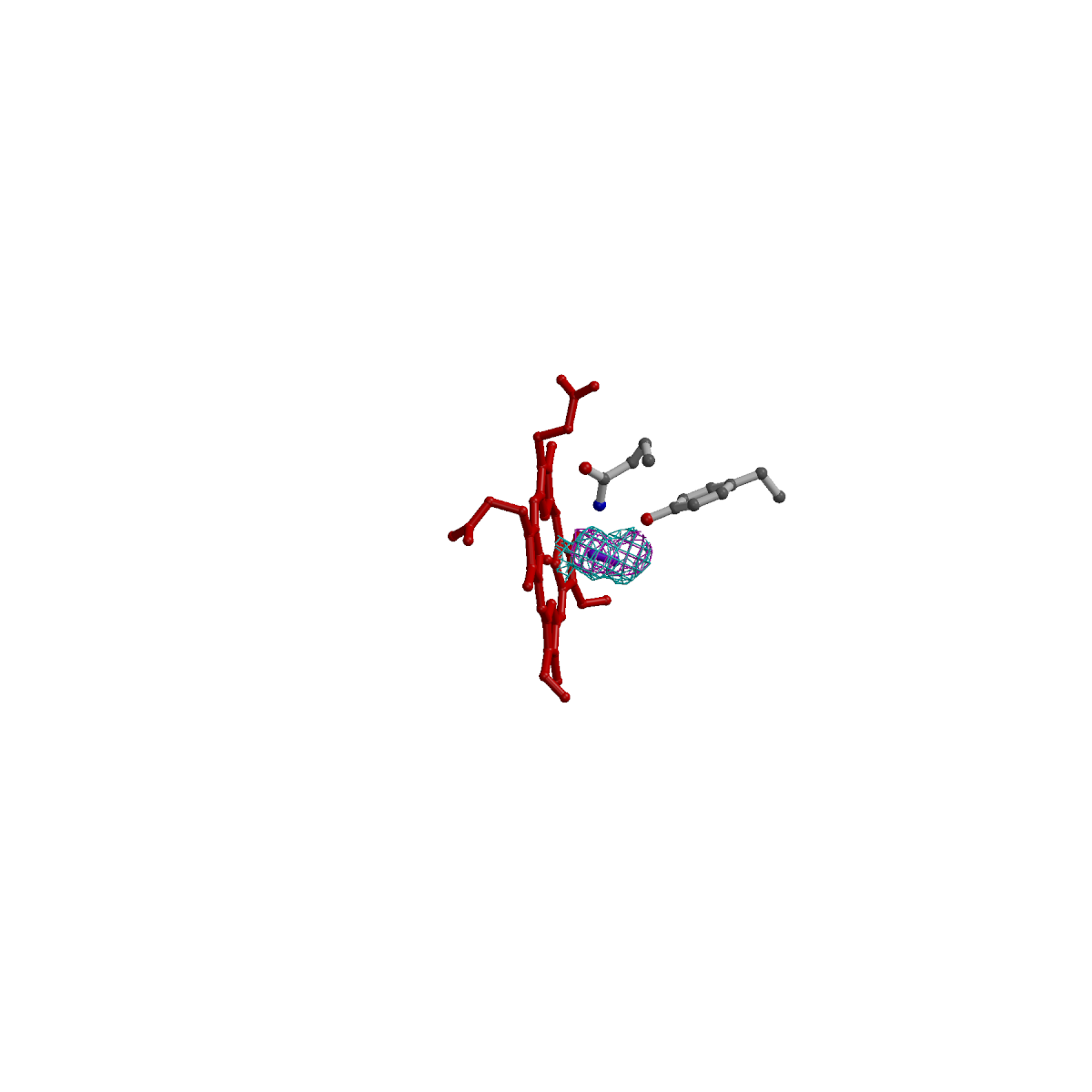

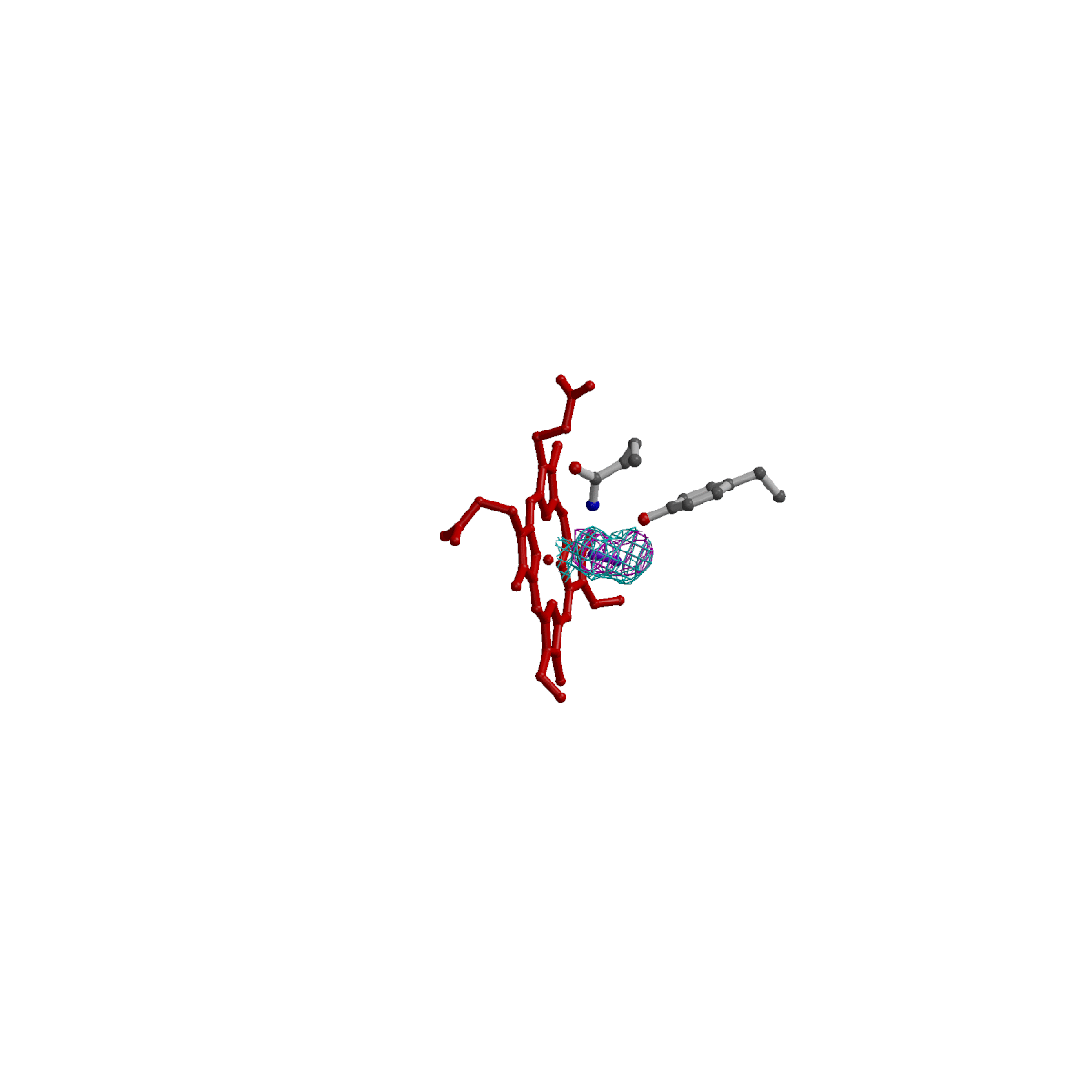


**B**


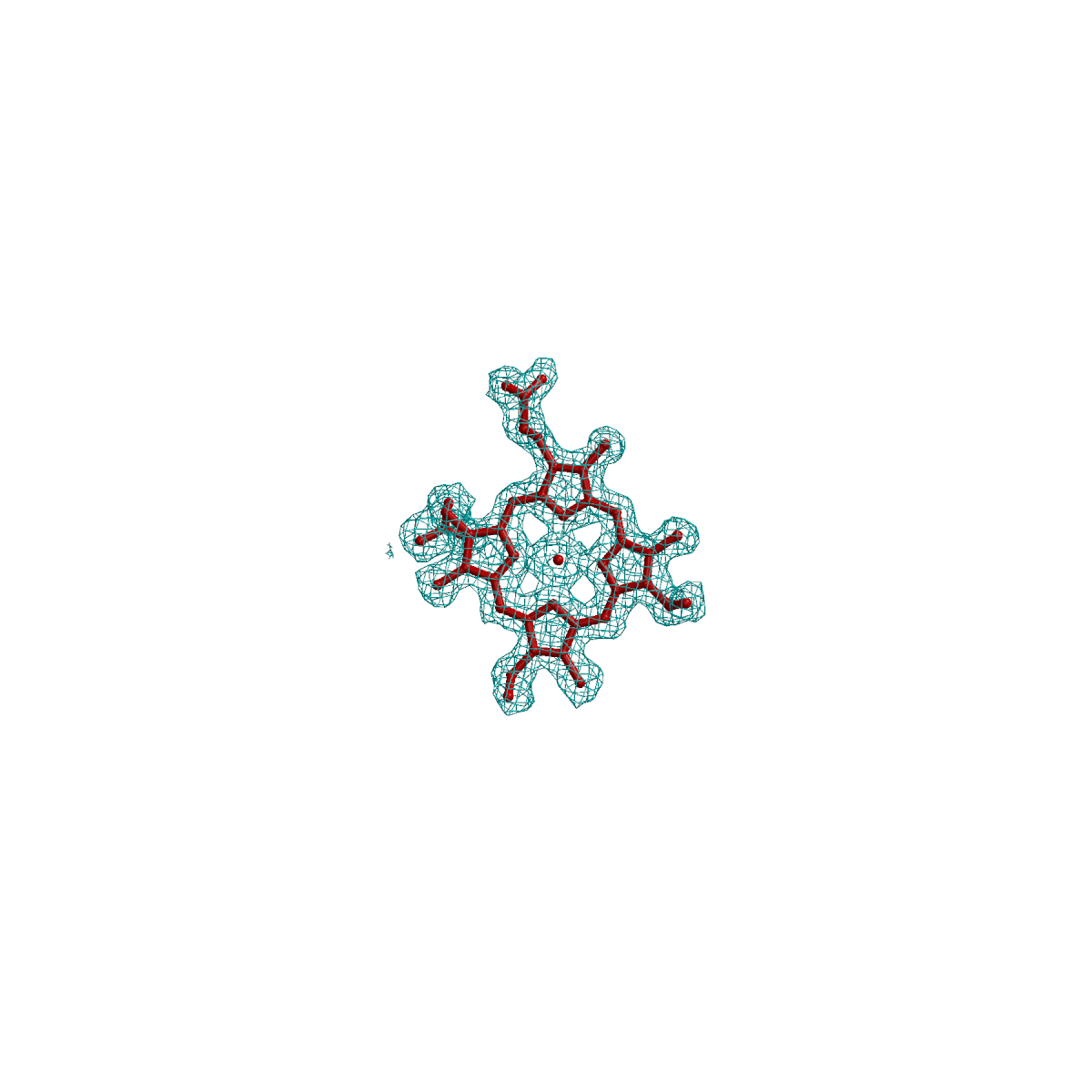

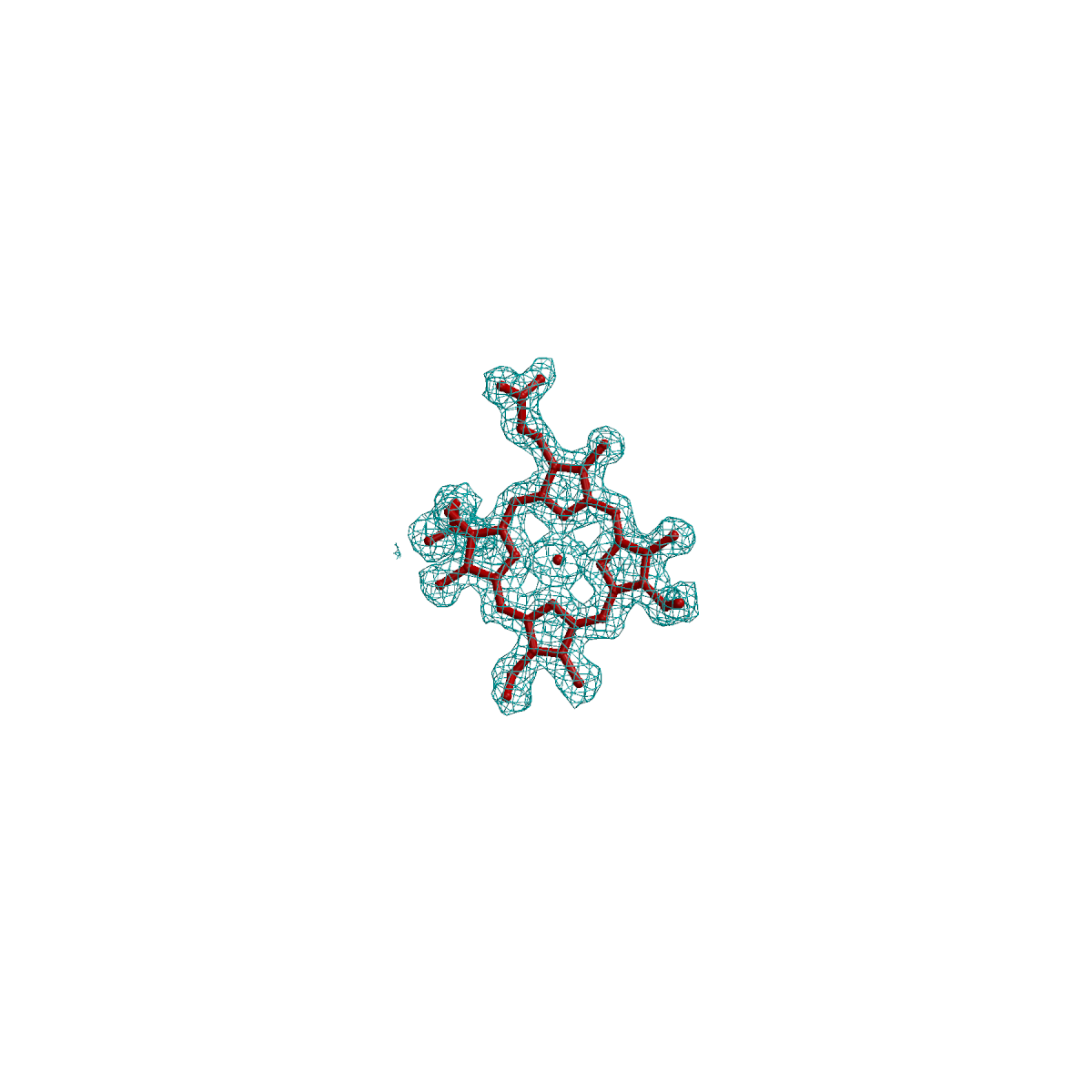


**Figure S2**: Stereo view of (A) heme distal site with the 2Fo-Fc and omit Fo-Fc electron density map (contoured at 1σ and 3σ, respectively) for oxygen shown as cyan and magenta mesh, respectively, confirming the presence of dioxygen coordinated to the heme group; (B) 2Fo-Fc electron density map (contoured at 1σ) for heme group highlights the high quality of map at 1.5 Å resolution.
