# Supplementary material for: Globin-like proteins in Caenorhabditis elegans: in vivo localization, ligand binding and structural properties
Source: BMC Biochem. 2010 Apr 2;11:17. doi: 10.1186/1471-2091-11-17 (PMC2867796; doi:10.1186/1471-2091-11-17)
Supplement: Additional file 3 — Globin expression levels under hypoxic conditions. Graph of globin expression levels under hypoxic conditions. [file 1471-2091-11-17-S3.DOC]

Additional file 3

Globin expression under hypoxic conditions

**Figure S3:** Expression levels of *C. elegans* globins *glb-26* and *glb-1* in young adult worms following 12h exposure to hypoxic conditions relative to normoxic conditions normalized to 3 reference genes. The expression ratios are the average values from 4 replicate cultures (biological repeats). Bars indicate the 95% confidence interval of the mean ** P<0.01
