# Supplementary material for: Globin-like proteins in Caenorhabditis elegans: in vivo localization, ligand binding and structural properties
Source: BMC Biochem. 2010 Apr 2;11:17. doi: 10.1186/1471-2091-11-17 (PMC2867796; doi:10.1186/1471-2091-11-17)
Supplement: Additional file 4 — Purification of Recombinant C. elegans globins. detailed protocol for the purification of all studied globins. [file 1471-2091-11-17-S4.DOC]

Additional file 4

Purification of Recombinant *C. elegans* globins

After expression, *C. elegans* globins, GLB-7, GLB-13, GLB-14, GLB-18, GLB-23 and GLB-29 (Table 1, Fig. 1) were localised as apo-protein in inclusion bodies. When no heme was incorporated into the *C. elegans* globins using the standard method, other methods were tried.

Cells were suspended in 50 mM Tris-HCl pH 8.5 mM EDTA, 1 mM phenylmethylsulfonylfluoride and exposed to three freeze-thaw cycles and sonication until complete lysis. Inclusion bodies were isolated by centrifugation at 8,000 g for 30 min and the pellet was suspended in 50 mM Tris-HCl pH 8, 5 mM EDTA, 2% deoxycholate and washed twice with this solution, followed by a final wash with water. The inclusion bodies were solubilised in 100 mM Tris pH 12, 2 M urea and incubated for 30 min at room temperature. A 1.4 molar excess of hemin was added and the pH was increased to pH 8.5 with 1 M NaOH. After 10 min incubation the samples were diluted 5 times with water, dialyzed against 50 mM Tris-HCl, pH 8.5, 150 mM NaCl, 0.5 mM EDTA and purified on a Sephacryl S200 gelfiltration column. Alternatively, inclusion bodies were solubilised in 6 M GuHCl, 100 mM Tris-HCl, pH 8, 72 mM DTT, and incubated for one hour at room temperature. After centrifugation (10 min at 10,000 g), the *C. elegans* globins were refolded by adding a 1.4 M excess of hemin and diluted 5 fold in 0.2 M KCl, 0.4 M arginine, 5 mM DTT, 2% glycine and 100 mM Tris-HCl pH 8 and dialysed against the same buffer.
